# Type 2 Diabetes Mellitus and *Helicobacter pylori* Gastritis in Patients Referred for Endoscopy—A Single-Center Romanian Study

**DOI:** 10.3390/life14091160

**Published:** 2024-09-13

**Authors:** Sabrina-Nicoleta Munteanu, Dragoș Huțanu, Ana-Maria Filip, Andreea Raluca Cozac-Szőke, Simona Mocan, Anca Negovan

**Affiliations:** 1 Department of Clinical Science-Internal Medicine, “George Emil Palade” University of Medicine, Pharmacy, Science, and Technology of Târgu Mureș, 540139 Mureș, Romania; sabrinamunteanu12@gmail.com (S.-N.M.); ancanegovan@yahoo.com (A.N.); 2 Pulmonology Department, Mureș County Clinical Hospital, Târgu Mureș, 540011 Mureș, Romania; 3 Internal Medicine Department, Emergency County Hospital of Targu Mures, 540136 Mureș, Romania; anna.maria121998@gmail.com; 4 Department of Pathophysiology, “George Emil Palade” University of Medicine, Pharmacy, Science, and Technology of Târgu Mureș, 540139 Mureș, Romania; szoke.andreea@yahoo.com; 5 Pathology Department, Mureș County Clinical Hospital, Târgu Mureș, 540011 Mureș, Romania; 6 Pathology Department, Emergency County Hospital of Targu Mures, 540136 Mureș, Romania; slmocan@yahoo.com

**Keywords:** type 2 diabetes mellitus, *Helicobacter pylori*, anemia

## Abstract

Background: Type 2 diabetes mellitus (T2DM) affects up to 10% of adults globally, and its complications can mask the risk of gastrointestinal bleeding or malignancy. Methods: Our study enrolled 633 endoscopic patients stratified according to T2DM presence (4:1 ratio in favor of the control group). Results: T2DM patients referred for endoscopy experienced lower prevalence of epigastric pain and heartburn (OR = 0.637/OR = 0.346, *p* < 0.05). Often being anemic (OR = 2.23, *p* < 0.001), they had significantly lower hemoglobin (*p* = 0.001) and serum iron (*p* = 0.02), but serum cholesterol was higher in non-diabetics. Ulcers, erosions and mucosal hemorrhages were comparable between groups (*p* < 0.05), although low-dose aspirin use was more prevalent in diabetics (*p* = 0.000, OR = 2.34). T2DM was associated with the increased frequency of antro-corporal active gastritis (OR = 1.451/OR 1.501), with smokers presenting a higher frequency of active *H. pylori* infection (OR = 3.37). T2DM predicted anemia (adjusted OR = 1.70) and the absence of gastroesophageal reflux symptoms (adjusted OR = 0.37), but not active *H. pylori* gastritis or premalignant lesions. Conclusion: In an endoscopic population, patients with T2DM had lower hemoglobin and serum iron levels. There was an inverse correlation between T2DM and heartburn. *H. pylori* gastritis and premalignant lesions occurred more frequently in diabetic patients (predominantly pangastritis) before adjusting for age or associated comorbidities, with smoking increasing the risk for active infection.

## 1. Introduction

Type 2 diabetes mellitus (T2DM) is considered to be the most widely spread endocrine disease, affecting up to 10% of the world’s adult population in most geographical areas. This metabolic condition has its foundation in relative insulin deficit, resistance to insulin or, in some cases, the combination of the two [1].

Its clinical importance lies in the possibility of complications developing, which can highly impact life expectancy. Diabetic neuropathy is a complication that can easily camouflage gastrointestinal (GI) changes, such as gastroesophageal reflux disease (GERD) symptoms (pyrosis), therefore increasing the risk for malignancies. Besides the well-known and studied vascular complications, T2DM’s implication in the pathology of the gastrointestinal tract has not been as thoroughly studied as it should be. Diabetic neuropathy has a substantial impact on the autonomous nervous system. This manifests through vagal implication, leading to the appearance of gastroparesis or gastric stasis. These changes can go unnoticed in most patients but can have a strong impact by causing severe vomiting and nausea, dysphagia, diarrhea, dyspepsia or abdominal pain [2].

The GI implications of T2DM are not limited to the alteration in motility but also involve an alteration in the immune response, with an increased risk of *Helicobacter pylori (H. pylori*) infection and bacterial colonization of the GI tract [1,2,3]. The role of routine endoscopy combined with histopathological screening is important in the evaluation of TD2M patients. Mucosal lesions occur when all the defense mechanisms fail to counteract the increased gastric acidity, the action of peptic enzymes or even extrinsic factors such as *H. pylori* infection, non-steroidal anti-inflammatory drugs (NSAIDs), smoking or alcohol consumption.

The identification of inflammatory signs in the gastric mucosa, when endoscopy with regular biopsies is performed, leads to the correct staging of gastritis induced by *H. pylori* persistent infection. Autoimmune gastritis can also be encountered when the immunomodulated loss of parietal cells takes place. The persistence of chronic gastritis can lead to gastric atrophy and intestinal metaplasia, both of which are strongly correlated with a risk of gastric adenocarcinoma [4].

*H. pylori* eradication therapy restores normal gastric mucosa or halts progression to mucosal lesions and reduce gastric cancer risk. A “test-and-treat” strategy for dyspepsia is recommended by all consensus conferences (for young patients without alarm symptoms), but antibiotic resistance is the main concern. The first-line recommended treatment in areas of high (>15%) or unknown clarithromycin resistance is bismuth quadruple therapy [5].

The purpose of this study was to define the impact of clinical predictors (underlying chronic diseases, digestive symptoms, changes in some routine laboratory parameters) on endoscopic and histopathological aspects in both antral and corporeal gastric mucosa in patients with T2DM to improve prevention strategies for bleeding and cancer risk.

## 2. Materials and Methods

### 2.1. Study Population

We performed a single-center retrospective case–control study that included 633 patients admitted to Medical Clinic No. 2 of the Emergency County Hospital of Targu Mures, Romania, over the course of 3 years (2019–2022). All patients underwent esophagogastroduodenoscopy and usual blood tests. Patients were hospitalized in the Medical Department for various conditions (cardiovascular, renal, respiratory or digestive), but EGD was indicated only in those with dyspeptic symptoms (epigastric pain/discomfort, pyrosis, belching, bloating, nausea/vomiting) or in the presence of alarming features (weight loss, anemia, loss of appetite). The frequency and severity of dyspeptic symptoms were assessed using a modified version of the Leeds Dyspepsia Questionnaire [6].

**Figure 1:**
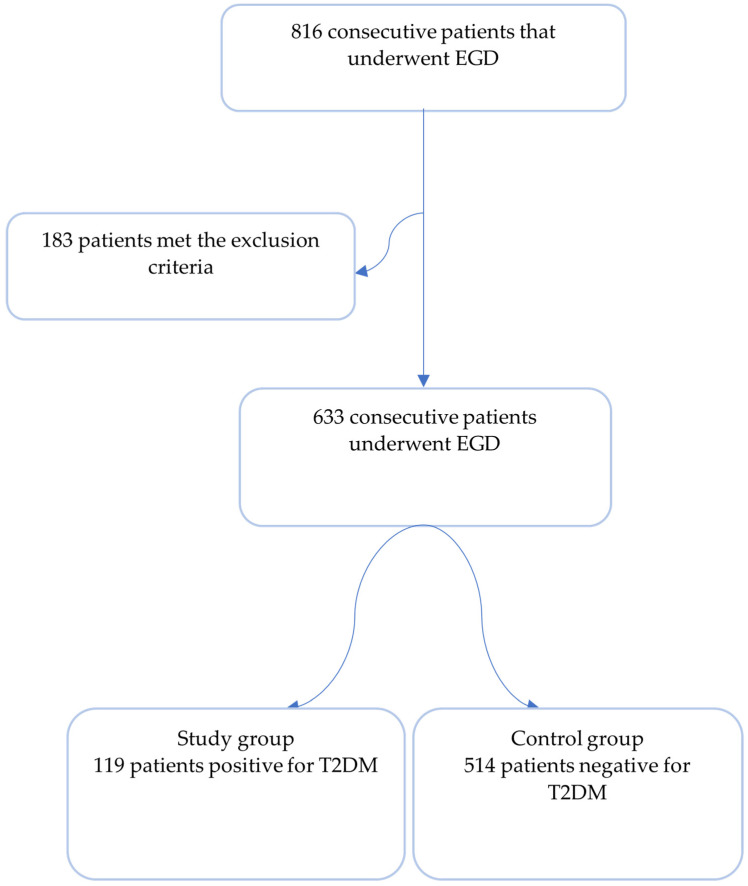
Patient selection process. Final included groups were composed of 119 patients with T2DM and 514 patients with no history of T2DM. EGD—esophagogastroduodenoscopy; T2DM—type 2 diabetes mellitus.

Of the 816 consecutive patients admitted to our clinic who underwent EGD procedures, 183 (22.4%) met at least one of the following exclusion criteria: age < 18 years, cancer diagnosis irrespective of localization, bleeding source (positive colonoscopy, positive fecal occult test), severe heart/renal diseases, dementia, inflammatory bowel disease, *H. pylori* eradication therapy, missing data referring to chronic comorbidities and drug use, social behavior or lack of consent regarding blood or gastric biopsy sampling. Inclusion criteria consisted of patients admitted to the clinic aged 18 or over who underwent EGD for dyspeptic or alarming features that agreed to participate in our study. We stratified patients based on the presence of a T2DM diagnosis in medical records or databases, obtaining a study group of 119 patients and a control group of 514 patients (Figure 1). We preferred using a control group with a 4:1 ratio to maximize the power of statistical significance.

### 2.2. Data Collection

We used direct interviews and medical records in order to gather data regarding chronic medications (including potential gastrotoxic drugs, e.g., non-steroidal anti-inflammatory drugs (NSAIDs), antiplatelet drugs (low-dose aspirin, clopidogrel and ticagrelor) and anticoagulants (low-weight molecular heparin, antivitamin K and direct oral anticoagulants) if they were used at least one month prior to the examination.

Comorbidities were recorded as follows: cardiovascular conditions (ischemic heart disease, congestive heart failure and valvulopathies), chronic kidney disease, osteoarticular diseases, respiratory disease (pulmonary fibrosis, asthma and chronic obstructive pulmonary disease) and cerebrovascular diseases (ischemic and hemorrhagic stroke).

The following cut-off laboratory values were proposed to be taken into consideration as normal ranges (according to the reference ranges proposed by our hospital): hemoglobin levels: 12–15 g/dL in women and 13–17 g/dL in men; mean corpuscular volume (MCV): 80–100 fL; serum iron reference range: 11.6–31.4 µmol/L; total cholesterol levels: <5.17 mmol/L; triglyceride levels < 1.7 mmol/L; fibrinogen: 2–4.0 g/dL; International Normalized Ratio (INR): 0.8–1.1.

The validity of our study may be limited across diverse populations due to the relatively small sample size of patients that were examined in a single center and presented similar social and demographic characteristics.

The study was approved by the Ethics Committee of the Emergency County Hospital of Targu Mures, Romania (decision number Ad. 32617 of 15 December 2022).

### 2.3. Endoscopic and Histologic Findings

Gastric lesions (erythema, erosions, submucosal hemorrhage and ulcers) were noted during endoscopy following a modified Lanza score version [7].

Five tissue biopsies were obtained according to the updated Sydney protocol (two antral, two corporal and one angular biopsy). The biopsies were sent in three containers (based on their biopsy site). Gastritis classification and grading were performed using the updated Sydney System protocol. After the fixed formalin biopsies were embedded in paraffin and 3–5 micron sections were processed, hematoxylin and eosin, modified Giemsa, and PAS-alcian blue were used to stain each biopsy sample.

**Figure 2:**
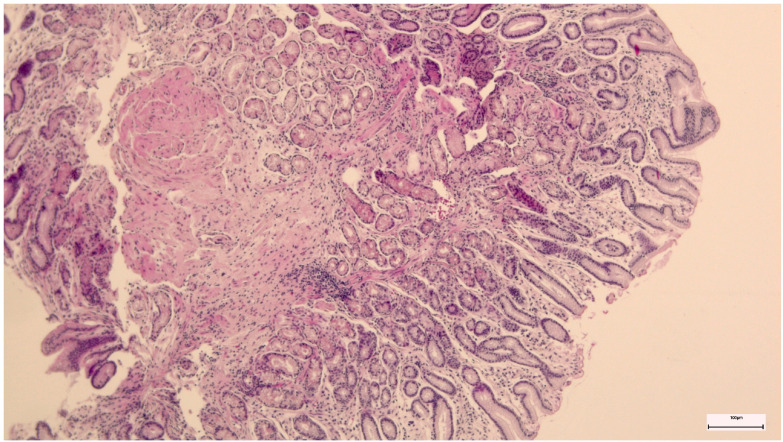
Antral reactive gastropathy.

A reactive gastropathy diagnosis was established if certain histologic changes, e.g., foveolar hyperplasia, mucin depletion in foveolar cells, fibromuscular replacement of the lamina propria with fibromuscular tissue and/or capillary congestion, were present (Figure 2).

**Figure 3:**
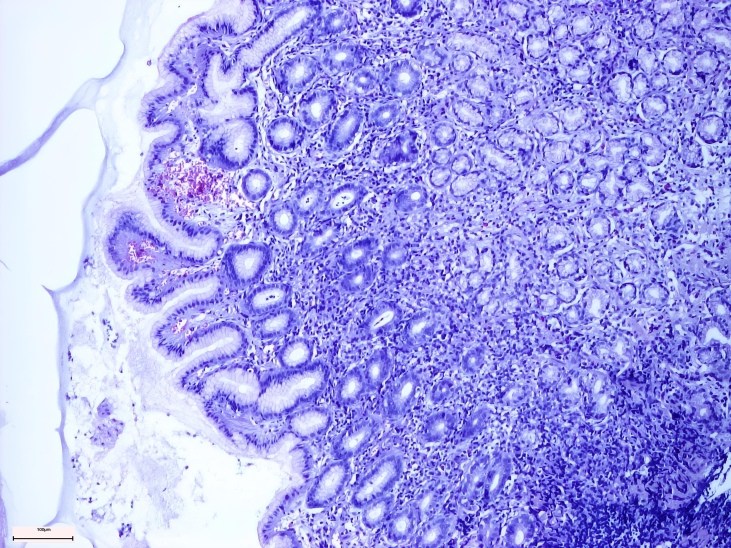
Antral active chronic gastritis.

**Figure 4:**
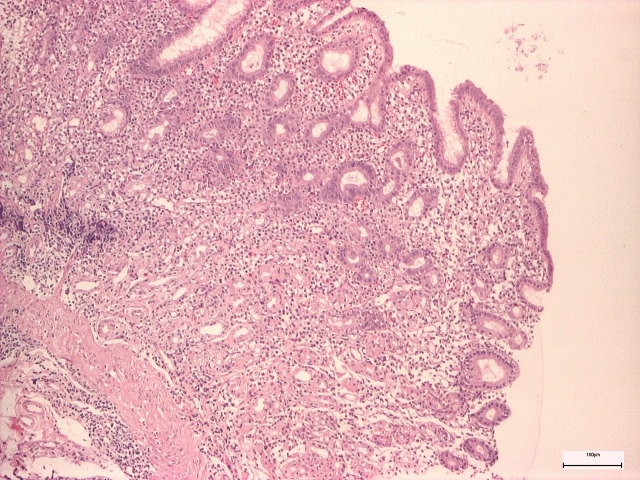
Corporeal active chronic gastritis.

Non-active gastritis was diagnosed if chronic inflammation (represented by the presence of mononuclear and plasma cells within the lamina propria, but not neutrophils) was present. Active gastritis diagnosis was established if acute inflammatory cells were present in corporal and antral mucosa (Figure 3 and Figure 4) [8].

### 2.4. Statistical Analysis

Statistical analysis was realized with IBM SPSS Statistics version 26.0.0, where the distribution of quantitative data was tested through histograms, Q-Q plots, and finally through the Shapiro–Wilk test for normality, confirming the presence of non-parametrical data. Therefore, all results referring to quantitative data were expressed as the median (minimum–maximum). Qualitative data were analyzed using frequencies, with results expressed in n (%). Differences between the two study groups were analyzed through the Mann–Whitney test for an independent sample and the Chi-square test, setting the significance limit at α = 0.05. This threshold was set accordingly with the study population size and international consensus. We also analyzed the odds ratio (OR) for the studied variables to evaluate the possible risks that T2DM may expose the patients to. Spearman correlations were also evaluated in order to identify possible connections between laboratory parameters and clinical and histopathological variables. Multivariate logistic regression was conducted, adjusting the effect of all variables that presented significance in order to quantify the prediction power of T2DM.

## 3. Results

The study group included a higher number of male patients (56.3%) compared to the control group (48.2%). Diabetic patients were significantly older than the control group (*p* = 0.000) (Table 1).

T2DM prevalence increases with age in endoscopic patients, with elderly patients (≥65 years) being 1.9 times more likely to have this condition. Epigastric pain/discomfort and heartburn were present more often in non-diabetic patients (46.3% and 24.3%), and both seemed to be negatively associated with T2DM (*p* = 0.038, OR 0.637 and *p* = 0.001, OR 0.346) (Table 2).

Hemoglobin and serum iron levels were significantly lower in diabetic patients (*p* = 0.001), while fibrinogen and INR values were higher in diabetic patients, reaching the threshold of statistical significance (Table 3). Cholesterol values were higher in non-diabetic patients (*p* = 0.000), while triglyceride values in T2DM patients were of lower value (*p* = 0.000).

Diabetic patients showed, as we expected, a much more frequent association with arterial hypertension (OR = 4.360, CI 95%: 2.615–7.268), valvulopathies (OR = 1.703, CI 95%: 1.118–2.593), ischemic heart disease (OR = 2.468, CI 95%: 1.639–3.715), heart failure (OR = 2.413, CI 95%: 1.604–3.631) and chronic kidney disease (OR = 4.555, CI 95%: 2.800–7.409), all with *p* < 0.001. Digestive, respiratory, autoimmune and neoplastic comorbidities showed similar distributions between the two groups (*p* > 0.05). Diabetic patients also showed a more frequent association with anemia (46.2% vs. 27,8%, OR = 2.230, CI 95%: 1.481–3.355, *p* < 0.001).

A significantly higher consumption of aspirin (39.5% vs. 21.8%), angiotensin-converting enzyme inhibitors (ACEIs—52.1% compared to 29%), and beta-blockers (68.9% vs. 44.2%) was noticed in diabetic patients. The use of direct oral anticoagulants (DOACs), vitamin K antagonists, clopidogrel or NSAIDs, as well as proton pump inhibitors (PPIs), was higher in diabetic patients, but not statistically significant (Table 4). 

Patients with T2DM did not present any statistically significant endoscopic changes/findings compared to non-diabetic patients (Table 5).

Regarding histopathological differences, a slight tendency toward the association of active antral and corporal gastritis (*p* = 0.08, OR = 1.451 and *p* = 0.054, OR = 1.501, respectively) with the presence of T2DM was identified, even though the threshold of statistical significance was not reached. The presence of *H. pylori* in biopsy samples was not statistically significant more prevalent in T2DM patients (*p* = 0.097, OR = 1.426) (Table 6).

Smokers showed a significantly higher frequency of *H. pylori* infection compared to non-smokers (OR = 3.37, CI 95%: 1.16–9.82, *p* = 0.04). However, no significant difference was identified between alcohol consumers and those who reported abstinence (*p* = 0.8798). 

Regarding laboratory values and the effect of these social behaviors on patients with T2DM, we identified mild but significant correlations using the Spearman correlation coefficient (r) between high alcohol consumption and values of mean corpuscular volume (MCV) and serum iron level. However, no correlation was found between high tobacco consumption and laboratory parameters (Table 7).

### Multivariate Regression Model

After adjusting for age, cardiovascular comorbidities (hypertension, valvulopathies, ischemic heart disease, heart failure) and aspirin consumption, T2DM remained an independent predictor for anemia (adjusted OR = 1.70, CI 95%: 1.10–2.64) and for higher low-dose aspirin consumption (adjusted OR = 1.62, CI 95%: 1.03–2.57). TD2M seems to be an independent predictor for the lack of GERD symptoms (heartburn) (adjusted OR = 0.37, CI 95%: 0.45–1.12), but not for epigastric pain. In our model, corporeal and antral chronic active gastritis or premalignant lesions were not associated with T2DM (Table 8).

## 4. Discussions

T2DM continues to show an upward trend in prevalence in the general population, emphasizing the need for increased attention to its gastroduodenal implications, including potential risks of bleeding and malignancy.

In the studied population, the consecutive patients who underwent endoscopy with and without T2DM were more frequently male, although the differences were not statistically significant.

Patients with T2DM and an indication for endoscopy have lower hemoglobin and serum iron levels, with anemia being the most common reason for recommending the procedure, with a tendency towards a higher prevalence of iron deficiency anemia, despite the similar frequency of mucosal lesions with non-diabetics identified in similar studies [9]. Our findings underline the importance of both clinical and biological follow-up in diabetic patients, who are more predisposed to accumulate hemorrhagic risk factors, such as antithrombotic therapy, the alteration of perception of gastroesophageal lesions, CKD, gastritis or *H. pylori* infection [10].

We identified the presence of T2DM as a “protective” factor against heartburn (confirmed by the multivariate regression analysis) and epigastric pain/discomfort. Alterations in perception and motility due to neuropathy involve an increased risk for complications while evolving asymptomatically, as studies have demonstrated that diabetic neuropathy is correlated with a significant increase in the risk of developing erosive esophagitis (OR = 5.01, CI: 1.40–17.95) [11]. The observations from our study draw attention to the importance of properly organizing screening programs for detecting associated structural changes, including neuropathy coexisting with multiple implications in the pathological chain and less obvious complications.

The significant differences identified in the total cholesterol values (4.09 vs. 4.695 mmol/L) may be due to the ongoing statin therapy in diabetic patients, and the higher levels of triglycerides (1.45 vs. 1.14 mmol/L) can be attributed to the failed management of diabetes. Dyslipidemia was proved to induce and maintain the systemic inflammation present in T2DM patients [12]. This condition also increases the risk of chronic kidney disease (CKD) (OR = 4.56, CI: 2.800–7.409), with approximately 32% of the diabetic patients included in the study having CKD [13]. Additionally, anemia was significantly more frequent in diabetic patients compared to non-diabetics (46.2% vs. 27.8%, *p* < 0.001) due to several mechanisms, including CKD and inflammation, with the difference being similar in different studied populations [14,15]. Due to the risk of T2DM in cardiovascular conditions, the differences between groups regarding the frequency of cardiovascular comorbidities or the consumption of aspirin, ACE inhibitors (ACEIs), and beta-blockers are not surprising.

No significant differences were identified between the two study groups for any of the evaluated changes or features on endoscopy. Hiatal hernia was identified as more frequent in the control group, similar to the study conducted by Regina Promberger et al., with the associated symptoms determining evaluation in these patients rather than other conditions [16].

The literature regarding the endoscopic and histopathological evaluation of patients with T2DM is not as widespread. Consequently, the associations between T2DM, *H. pylori* infection and their mucosal and systemic consequences are not yet well defined [17]. In our study, we identified an increased frequency of gastric mucosal active antral and corporeal gastritis (OR: 1.451, 95% CI: 0.955–2.204 and OR: 1.501, 95% CI: 0.991–2.272, respectively), with *H. pylori* identified in regular biopsy samples more frequently (OR: 1.426, 95% CI: 0.937–2.171) in diabetic patients compared to the control group, although they remained above the threshold of statistical significance. A meta-analysis that included 41 studies demonstrated a positive association between *H. pylori* infection and diabetes mellitus [18], but there are various studies from Romania [19] and Japan [20] that also did not identify statistically significant differences in the diabetic vs. non-diabetic populations.

The presence of premalignant gastric lesions (glandular atrophy and intestinal metaplasia) was also comparable in the diabetic and non-diabetic endoscopic populations [21]. However, after applying the multivariate regression model, T2DM did not remain a predictor for active *H. pylori* infection, nor for the presence of premalignant GI lesions. After adjusting for comorbidities, aspirin consumption and age, T2DM remained an independent predictor for anemia, suggesting its high relevance in the clinical setting, with many conditions as predictors for its development, not only digestive lesions [22]. Except for the decreased frequency of heartburn, T2DM does not seem to impact the complaints of patients referred for endoscopy. Asymptomatic gastroesophageal reflux disease can occur in patients with T2DM, leading to esophageal mucosa damage. If future research confirms that T2DM reduces the incidence and severity of heartburn by masking this symptom, this could impact screening and treatment protocols, highlighting the importance of proactive gastroesophageal reflux prevention in diabetic patients, even in the absence of typical symptoms.

As our results suggest, clinicians should consider routine screening for gastroesophageal reflux disease even when typical symptoms such as heartburn are not present. Moreover, routine screening procedures for anemia and chronic kidney disease should be provided more often in these patients in order to prevent their complications. In those who smoke, *H. pylori* screening and eradication should be prioritized. Endoscopic monitoring should be recommended in patients that present premalignant gastric lesions and in those treated with low-dose aspirin due to the higher risk of gastrointestinal bleeding.

The association between diabetes and upper digestive diseases should remain open to further research to improve the quality of life and minimize the potential for bleeding and malignant progression. Our study identified the importance of clinical predictors in upper endoscopic and pathological findings in diabetic patients investigated on endoscopy, emphasizing the lack of symptoms in these patients and the possible impact of metabolic control and systemic inflammation on disease progression and complications. The study demonstrated the importance of an interdisciplinary approach toward the risk factors for digestive complications in an aging populations’ accumulated comorbidities, with T2DM as a background condition.

## 5. Conclusions

Patients with diabetes underwent endoscopic investigation for anemia, rather than for digestive symptoms. No significant differences were found regarding endoscopic lesions in diabetic patients compared with non-diabetic, while heartburn is significantly negatively correlated with diabetes in endoscopic population. Epigastric pain is less frequent in patients with T2DM, but they experience more frequent nausea and vomiting. *H. pylori* gastritis and premalignant gastric lesions had a higher frequency in T2DM patients referred for endoscopy, with a pangastritis phenotype predominance, but not after adjusting for age and presence of associated comorbidities. Smoking is associated with *H. pylori* infection in diabetic patients, but alcohol does not influence the association. Further research into the overlap of T2DM and *H. pylori* infection is needed to confidently predict and acknowledge possible precautions and solutions.

## Figures and Tables

**Table 1:** Demographic differences among T2DM and non-diabetic patients.

Variables.	Study Group T2DM+ (n = 119)	Control Group T2DM− (n = 514)	*p*
**Age**	**68 (37–86)**	**63 (23–92)**	**0.000 ^a^**
Sex—n (%)			
Male	67 (56.3)	248 (48.2)	0.113 ^b^

^a^: Mann–Whitney test; ^b^: Chi-square test; T2DM: type 2 diabetes mellitus.

**Table 2:** Group differences regarding clinical features among T2DM and non-diabetic patients.

Symptoms—n (%).	Study Group T2DM+ (n = 119)	Control Group T2DM− (n = 514)	*p* ^b^	OR ^c^	CI 95% ^d^
**Epigastric pain/discomfort**	**39 (35.5)**	**238 (46.3)**	**0.038**	**0.637**	**0.415–0.977**
**Heartburn**	**11 (10)**	**125 (24.3)**	**0.001**	**0.346**	**0.180–0.665**
Belching	3 (2.7)	18 (3.5)	0.683	0.773	0.224–2.670
Bloating	16 (14.5)	94 (18.3)	0.350	0.761	0.428–1.352
Nausea/vomiting	28 (25.5)	99 (19.3)	0.143	1.431	0.884–2.317
Diarrhea	5 (4.5)	30 (5.8)	0.593	0.768	0.291–2.027
Weight loss	10 (9.1)	72 (14)	0.166	0.614	0.306–1.231
Loss of appetite	9 (8.2)	66 (12.8)	0.173	0.605	0.292–1.254

^b^: Chi-square test; ^c^: odds ratio; ^d^: confidence interval; T2DM: type 2 diabetes mellitus.

**Table 3:** Distribution of laboratory findings among T2DM and non-diabetic patients.

Laboratory Results	Study Group T2DM+ (n = 119)	Control Group T2DM− (n = 514)	*p* ^a^
**Hemoglobin (g/dL)**	**12.3 (4.7–18.1)**	**13.2 (3.6–18)**	**0.001**
Mean Corpuscular Volume (femtoliters)	86.2 (62.2–115.7)	86.3 (52.2–124.1)	0.098
**Serum Iron (micromoles/L)**	**11.6 (1.6–49.7)**	**13.05 (1–61.4)**	**0.02**
**Fibrinogen (g/dL)**	**4.1 (1.1–8.2)**	**3.5 (1.2–17.2)**	**0.000**
**INR**	**1.12 (0.78–6.73)**	**1.05 (0.84–4.71)**	**0.000**
**Cholesterol (mmol/L)**	**4.09 (1.08–7.74)**	**4.69 (1.67–12.55)**	**0.000**
**Triglycerides (mmol/L)**	**1.45 (0.44–4.89)**	**1.14 (0.04–5.40)**	**0.000**

^a^: Mann–Whitney test; T2DM: type 2 diabetes mellitus INR: International Normalized Ratio.

**Table 4:** Group differences regarding chronic medication use among T2DM and non-diabetic patients.

Medication—n (%)	Study Group T2DM+ (n = 119)	Control Group T2DM− (n = 514)	*p* ^b^	OR ^c^	CI 95% ^d^
NSAIDs	14 (11.8)	97 (18.9)	0.066	0.573	0.315–1.044
Aspirin	**47 (39.5)**	**112 (21.8)**	**0.000**	**2.343**	**1.535–3.577**
ACEIs	**62 (52.1)**	**149 (29.0)**	**0.000**	**2.665**	**1.774–4.003**
PPIs	77 (64.7)	299 (58.2)	0.191	1.318	0.871–1.996
Vitamin K Antagonists	15 (12.6)	44 (8.6)	0.171	1.541	0.826–2.873
DOACs	14 (11.8)	34 (6.6)	0.056	1.882	0.976–3.632
Clopidogrel	18 (15.1)	64 (12.5)	0.434	1.253	0.712–2.206
Beta-blockers	**82 (68.9)**	**227 (44.2)**	**0.000**	**2.802**	**1.831–4.288**

^b^: Chi-square test; ^c^: odds ratio; ^d^: confidence interval; T2DM: type 2 diabetes mellitus; NSAIDs: non-steroidal anti-inflammatory drugs; DOAC: new oral anticoagulant; ACEIs: angiotensin-converting enzyme inhibitors; PPIs: proton pump inhibitors.

**Table 5:** Group differences regarding endoscopic findings among T2DM and non-diabetic patients.

Endoscopic Findings—n (%)	Study Group T2DM+ (n = 119)	Control Group T2DM− (n = 514)	*p* ^b^	OR ^c^	CI 95% ^d^
Gastric resection	2 (1.7)	23 (4.5)	0.158	0.365	0.085–1.570
Hiatal hernia	30 (25.2)	165 (32.1)	0.142	0.713	0.453–1.122
*Gastric erythema*					
antral	90 (75.6)	413 (80.4)	0.251	0.759	0.474–1.216
corporal	26 (21.8)	110 (21.4)	0.915	1.027	0.633–1.665
*Gastric erosions*					
antral	36 (30.3)	161 (31.3)	0.820	0.951	0.617–1.467
corporal	7 (5.9)	33 (6.4)	0.828	0.911	0.393–2.113
Gastric ulcers (irrespective of location)	9 (7.6)	32 (6.2)	0.593	1.232	0.572–2.656
*Submucosal hemorrhage*					
antral	7 (5.9)	39 (7.6)	0.518	0.761	0.332–1.747
corporal	15 (12.6)	43 (8.4)	0.149		

^b^: Chi-square test; ^c^: odds ratio; ^d^: confidence interval; T2DM: type 2 diabetes mellitus.

**Table 6:** Histopathological differences among T2DM and non-diabetic patients.

Histologic Findings—n (%)	Study Group T2DM+ (n = 119)	Control Group T2DM− (n = 514)	*p* ^b^	OR ^c^	CI 95% ^d^
*Reactive gastropathy*					
antral	39 (32.8)	190 (37)	0.372	0.831	0.545–1.269
corporal	13 (10.9)	54 (10.5)	0.894	1.045	0.550–1.984
*Chronic inactive gastritis*					
antral	21 (17.6)	91 (17.9)	0.814	0.983	0.583–1.657
corporal	20 (16.8)	96 (18.7)	0.635	0.880	0.518–1.493
*Chronic active gastritis*					
antral	44 (37)	148 (28.8)	0.080	1.451	0.955–2.204
corporeal	46 (38.7)	152 (29.6)	0.054	1.501	0.991–2.272
*Premalignant gastric lesions*					
Glandular atrophy	31 (26.1)	124 (24.1)	0.660	1.108	0.702–1.749
Intestinal metaplasia	42 (35.3)	170 (33.1)	0.644	1.104	0.726–1.677
*H. Pylori* infection	43 (36.1%)	146 (28.4%)	0.097	1.426	0.937–2.171

^b^: Chi-square test; ^c^: odds ratio; ^d^: confidence interval; T2DM: type 2 diabetes mellitus.

**Table 7:** Correlation between laboratory findings and alcohol/tobacco consumption in T2DM patients.

Laboratory Parameters/Social Behaviors (r, *p*)	Alcohol	Tobacco
Hemoglobin (g/dL)	r = 0.30, *p* = 0.799	r = −0.189, *p* = 0.076
MCV (fL)	**r = 0.228, *p* = 0.049**	r = −0.097, *p* = 0.367
Serum Iron (μmol/L)	**r = 0.242, *p* = 0.036**	r = −0.094, *p* = 0.383
Fibrinogen (g/dL)	r = −0.104, *p* = 0.375	r = 0.021, *p* = 0.843
INR	r = 0, *p* = 1.000	r = 0.123, *p* = 0.253

T2DM: type 2 diabetes mellitus; INR: International Normalized Ratio.

**Table 8:** Multivariate logistic regression model.

Parameter	B ^a^	SE ^b^	*p* ^c^	Adjusted OR ^d^	CI 95% ^e^
Epigastric pain	−0.34	0.23	0.14	0.71	0.45–1.12
Heartburn	−0.98	0.35	**<0.01**	**0.37**	**0.18–0.75**
Anemia	0.53	0.22	**<0.01**	**1.70**	**1.10–2.64**
*H. pylori* infection	0.42	0.22	0.06	1.52	0.98–2.37
Antral chronic active gastritis	0.34	0.22	0.13	1.41	0.90–2.20
Corporeal chronic active gastritis	0.42	0.22	0.06	1.53	0.99–2.36
Intestinal metaplasia	−0.03	0.22	0.88	0.96	0.62–1.50
Glandular atrophy	−0.05	0.24	0.84	0.95	0.58–1.54

^a^—coefficient; ^b^—standard error; ^c^—Wald test; ^d^—odds ratio; ^e^—confidence interval.

## Data Availability

The dataset is available on request from the authors.

## References

[1] Kumar P., Clark M. (2021). Kumar and Clark’s Clinical Medicine.

[2] Buddam A., Hoilat G.J., Dacha S. (2022). Gastric Stasis.

[3] Nodoushan S.H., Nabavi A. (2019). The Interaction of *Helicobacter pylori* Infection and Type 2 Diabetes Mellitus. Adv. Biomed. Res..

[4] Raza M., Bhatt H. (2024). Atrophic Gastritis.

[5] Malfertheiner P., Megraud F., Rokkas T., Gisbert J.P., Liou J.-M., Schulz C., Gasbarrini A., Hunt R.H., Leja M., O’Morain C. (2022). Management of *Helicobacter pylori* infection: The Maastricht VI/Florence consensus report. Gut.

[6] Fraser A., Delaney B.C., Ford A.C., Qume M., Moayyedi P. (2007). The Short-Form Leeds Dyspepsia Questionnaire validation study. Aliment. Pharmacol. Ther..

[7] Lanza F.L., Collaku A., Liu D.J. (2018). Endoscopic comparison of gastroduodenal injury with over-the-counter doses of new fast-dissolving ibuprofen and paracetamol formulations: A randomized, placebo-controlled, 4-way crossover clinical trial. Clin. Exp. Gastroenterol..

[8] Yakirevich E., Resnick M.B. (2013). Pathology of Gastric Cancer and Its Precursor Lesions. Gastroenterol. Clin. N. Am..

[9] Pradeepa R., Shreya L., Anjana R.M., Jebarani S., Raj N.K., Kumar M.S., Jayaganesh P., Swami O.C., Mohan V. (2022). Frequency of iron deficiency anemia in type 2 diabetes—Insights from tertiary diabetes care centres across India. Diabetes Metab. Syndr. Clin. Res. Rev..

[10] Boehme M.W.J., Autschbach F., Ell C., Raeth U. (2007). Prevalence of silent gastric ulcer, erosions or severe acute gastritis in patients with type 2 diabetes mellitus—A cross-sectional study. Hepato-Gastroenterol..

[11] Lee S.D., Keum B., Chun H.J., Bak Y.T. (2011). Gastroesophageal reflux disease in type II diabetes mellitus with or without peripheral neuropathy. J. Neurogastroenterol. Motil..

[12] Mahemuti N., Jing X., Zhang N., Liu C., Li C., Cui Z., Liu Y., Chen J. (2023). Association between Systemic Immunity-Inflammation Index and Hyperlipidemia: A Population-Based Study from the NHANES (2015–2020). Nutrients.

[13] Thomas M.C., Cooper M.E., Zimmet P. (2016). Changing epidemiology of type 2 diabetes mellitus and associated chronic kidney disease. Nat. Rev. Nephrol..

[14] Junaid O.A., Ojo O.A., Adejumo O.A., Junaid F.M., Owolade S.S., Ojo O.E., Kolawole B.A., Ikem T.R. (2022). Prevalence of cardiovascular risk factors and their association with renal impairment in elderly with type 2 diabetes mellitus patients in a Nigerian tertiary hospital: A cross-sectional study. Pan Afr. Med. J..

[15] Sajid A., Waseem S. (2020). Study of anemia in diabetic and non-diabetic subjects: A hospital-based study in Lucknow, Uttar Pradesh. Natl. J. Physiol. Pharm. Pharmacol..

[16] Promberger R., Lenglinger J., Riedl O., Seebacher G., Eilenberg W.H., Ott J., Riegler F.M., Gadenstätter M., Neumayer C. (2013). Gastro-oesophageal reflux disease in type 2 diabetics: Symptom load and pathophysiologic aspects—A retro-pro study. BMC Gastroenterol..

[17] Valenzano M., Bisio A., Grassi G. (2019). *Helicobacter pylori* and diabetes mellitus: A controversial relationship. Minerva Endocrinol..

[18] Mansori K., Moradi Y., Naderpour S., Rashti R., Moghaddam A.B., Saed L., Mohammadi H. (2020). *Helicobacter pylori* infection as a risk factor for diabetes: A meta-analysis of case-control studies. BMC Gastroenterol..

[19] Ciortescu I., Sfarti C., Stan M., Graur M., Stanciu C. (2009). Prevalence of *Helicobacter pylori* infection in patients with diabetes mellitus. Rev. Med. Chir. Soc. Med. Nat. Iasi.

[20] Tamura T., Morita E., Kawai S., Sasakabe T., Sugimoto Y., Fukuda N., Suma S., Nakagawa H., Okada R., Hishida A. (2015). No association between *Helicobacter pylori* infection and diabetes mellitus among a general Japanese population: A cross-sectional study. SpringerPlus.

[21] Aumpan N., Vilaichone R.-K., Pornthisarn B., Chonprasertsuk S., Siramolpiwat S., Bhanthumkomol P., Nunanan P., Issariyakulkarn N., Ratana-Amornpin S., Miftahussurur M. (2021). Predictors for regression and progression of intestinal metaplasia (IM): A large population-based study from low prevalence area of gastric cancer (IM-predictor trial). PLoS ONE.

[22] Munteanu S.N., Cozac-Szőke A.R., Mocan S., Zait T.M., Rus R.I., Petri R.E., Negovan A. (2023). Predictors of anemia without active bleeding signs in patients referred for endoscopy. Acta Marisiensis Ser. Medica.

